# Impact of an integrated hospital-community care pathway on the management of venous thromboembolism: the venous thromboembolic disease care pathway 93 study

**DOI:** 10.1016/j.rpth.2026.106956

**Published:** 2026-09-12

**Authors:** Jérémy Datchanamourty, Antonio Morabito, Raphael Lhote, Siwar Hammami, Nanthara Sritharan, Matthieu Plichart, Sylvain Le Jeune

**Affiliations:** 1 Department of Vascular Medicine, Avicenne Hospital, Assistance Publique–Hôpitaux de Paris, Bobigny, France; 2 Department of Research, Avicenne Hospital, Assistance Publique–Hôpitaux de Paris, Bobigny, France

**Keywords:** anticoagulation, care coordination, care pathway, loss to follow-up, transition of care, venous thromboembolism

## Abstract

**Background:**

Venous thromboembolism (VTE) carries a high risk of recurrence and bleeding complications. Care transitions represent a vulnerable period, particularly in medically underserved areas where loss to follow-up is common. Integrated hospital-community care pathways may improve outcomes, but data are limited.

**Objectives:**

To evaluate whether an integrated hospital–community care pathway for patients with venous thromboembolism was associated with improved clinical outcomes and continuity of care compared with standard care.

**Methods:**

We conducted a single-center, retrospective, observational study of 161 consecutive patients managed for VTE in the day hospital at Avicenne Hospital (Bobigny, France) from December 2022 to December 2024. Patients enrolled in a structured city hospital care pathway (*n* = 64) were compared with those receiving standard care (*n* = 97). The primary endpoint was a composite of thrombotic recurrence, bleeding, hospitalization, or death. Inverse probability of treatment weighting was used to address confounding.

**Results:**

The structured pathway was associated with a lower risk of the primary composite endpoint (hazard ratio [HR], 0.44; 95% CI, 0.21-0.93; *P* = .03), consistent with the inverse probability of treatment weighting analysis (HR, 0.44; 95% CI, 0.21-0.92; *P* = .03). Loss to follow-up was markedly lower in the structured pathway group (7.8% vs 30%; *P* = .002), and patients lost to follow-up had a 3.5-fold higher risk of complications (HR, 3.50; 95% CI, 1.71-7.18; *P* < .001). Enrollment in the pathway was associated with lower odds of loss to follow-up (odds ratio, 0.21; 95% CI, 0.07-0.53; *P* = .002).

**Conclusion:**

In this single-center, retrospective cohort study, a structured city hospital care pathway was associated with a lower risk of composite complications and a lower loss-to-follow-up rate among patients managed for VTE. These findings support the potential value of integrated care coordination pathways, particularly in medically underserved areas.

## Essentials

• Care transition after venous thromboembolism remains vulnerable.
• We compared a structured care pathway with standard care.
• The pathway was associated with fewer complications and less loss to follow-up.
• Structured coordination may improve continuity of care.

## Graphical abstract

**Figure:**
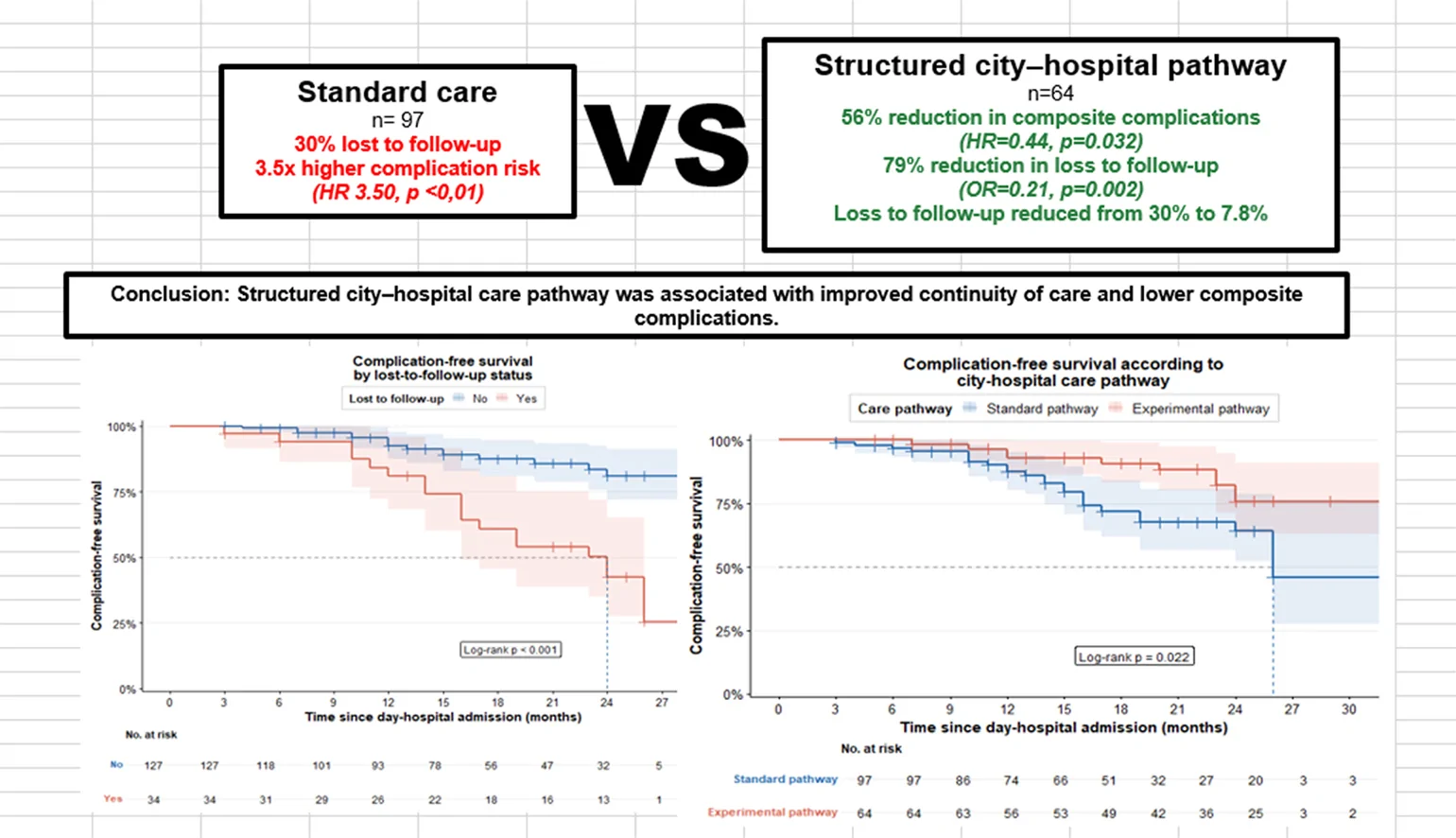

## 1 Introduction

Venous thromboembolism (VTE), which includes deep vein thrombosis (DVT) and pulmonary embolism (PE), is a major cause of morbidity and mortality worldwide and represents the third leading cause of cardiovascular death [1]. Its incidence, estimated at 1 to 2 cases per 1000 person-years in Western countries, increases significantly with age, reflecting the impact of population aging and comorbidities [2].

In France, VTE remains a significant public health issue, with >62,000 annual hospitalizations and ∼900,000 patients living with VTE [3]. Despite recent therapeutic advances, particularly the introduction of direct oral anticoagulants (DOACs), 1-year mortality remains high at around 20%, highlighting the limitations of current strategies [3].

VTE is not limited to an acute event but extends into a chronic disease characterized by a significant risk of recurrence and long-term, debilitating complications for patients, such as postthrombotic syndrome and chronic thromboembolic pulmonary hypertension. Its complex pathophysiology is based on the interaction among venous stasis, hypercoagulability, and endothelial dysfunction, integrated into Virchow’s triad [1]. The accumulation of risk factors, whether transient or permanent, contributes to the variability of risk and complicates management.

Anticoagulant therapy, an essential component of treatment, is associated with a delicate balance between antithrombotic efficacy and bleeding risk. In this context, care transitions, particularly hospital discharge, represent periods of high risk for adverse events, linked to coordination failures, medication errors, and insufficient adherence [3,4].

Thus, improving care continuity and optimizing coordination among different healthcare providers appear to be major levers for improving the prognosis of patients with VTE.

Integrated hospital-community care pathways, aimed at structuring follow-up and strengthening coordination among healthcare providers, could address these challenges, particularly for patients in precarious situations and in areas with limited medical resources.

The widespread use of DOACs has substantially simplified VTE management compared with the vitamin K antagonist (VKA) era, during which regular biological monitoring and frequent medical contact were required. However, this simplification may also reduce opportunities for structured follow-up, medication reconciliation, and early identification of complications. Whether reduced follow-up intensity after acute VTE has clinical consequences remains insufficiently studied, particularly in medically underserved areas where continuity of care (COC) is challenging.

In this context, the Parcours Maladie Thromboembolique Veineuse 93 study was designed to assess the association between a structured care pathway involving multiple community and hospital healthcare professionals and clinical events and continuity of follow-up in patients treated for VTE.

## 2 Methods

### 2.1 Study design and population

We conducted a single-center, retrospective, observational study at the Vascular Medicine Day Hospital, Avicenne Hospital (Bobigny, France). Patients were identified at the time of management at the Vascular Medicine Day Hospital after diagnosis of VTE and initiation of anticoagulant therapy. Consecutive patients managed between December 1, 2022, and December 31, 2024, were included if follow-up data were available. Patients were divided into 2 groups: those enrolled in a structured city hospital care pathway (structured pathway group) and those receiving standard care (standard care group). Patients without available clinical follow-up data were excluded.

Race and ethnicity data were not collected because these variables are not routinely recorded in French clinical practice and their systematic collection is restricted by French regulations.

### 2.2 Organization of the city hospital care pathway

The structured city hospital care pathway was implemented progressively in collaboration with local Communautés Professionnelles Territoriales de Santé (CPTS; Territorial Professional Health Communities). CPTS are multidisciplinary primary care organizations established in France to strengthen coordination between hospital and community healthcare providers. They bring together general practitioners, community nurses, pharmacists, physiotherapists, and other allied health professionals working in the same geographic area. Their mission is to improve COC, facilitate communication among healthcare professionals, organize patient care pathways, and improve access to healthcare.

Patients were enrolled in the structured pathway only if their place of residence was covered by a collaborating CPTS. During the implementation phase, some geographic areas had no operational collaborating CPTS, while others had not yet joined the project despite repeated contact. Consequently, these patients received standard care. Allocation therefore depended on the geographic availability and operational status of the local CPTS rather than patients’ clinical characteristics.

### 2.3 The city hospital care pathway

After inclusion in the structured pathway, a dedicated care coordinator organized patients’ follow-up before hospital discharge. The coordinator verified whether each patient had an identified primary care physician. When no general practitioner had been identified, the coordinator actively facilitated identification of one whenever possible. Follow-up appointments were scheduled at 1, 3, 9, and 12 months, with vascular medicine follow-up at 6 and 12 months according to clinical need, and home nursing visits were arranged when required to assess treatment adherence, bleeding risk, and fall risk.

The coordinator also informed the community pharmacist about the anticoagulant treatment and facilitated communication between hospital physicians and community healthcare professionals. Community providers could directly contact the hospital vascular medicine team for rapid advice in case of complications, adherence concerns, or questions regarding anticoagulant management.

### 2.4 Standard care

Patients allocated to the standard care group received routine management according to usual clinical practice. Before discharge, they received anticoagulant treatment and standard discharge recommendations. Patients were advised to consult their general practitioner after discharge and to attend a vascular medicine follow-up visit within 3 to 6 months according to routine practice. However, no dedicated care coordinator was involved; follow-up appointments were not systematically organized or verified before discharge, and no structured communication was established with community pharmacists or home care nurses.

Unlike the structured city hospital care pathway, standard care did not include systematic medication reconciliation with community healthcare professionals, active organization of primary care follow-up, or dedicated coordination between hospital and community providers.

### 2.5 Anticoagulant management

Anticoagulant therapy was prescribed in accordance with current international guidelines and individual patient characteristics. DOACs were used as first-line treatment when appropriate. Low-molecular-weight heparin was mainly prescribed for patients with active cancer (*n* = 6) and for those during pregnancy (*n* = 4). VKAs were reserved for specific clinical situations, including antiphospholipid syndrome (*n* = 7), severe obesity (*n* = 4), recurrent VTE while receiving a DOAC (*n* = 3), and clinically significant drug interactions precluding DOAC use (*n* = 3).

### 2.6 Study objectives and endpoints

The primary objective was to assess the association between the structured city hospital care pathway and the risk of complications. The primary endpoint was the incidence of combined complications, defined as thrombotic recurrence, bleeding events, hospitalization, or death during follow-up. Secondary objectives included comparing complication rates among patients lost to follow-up according to pathway allocation, comparing complication rates by loss-to-follow-up status, and assessing the association between the pathway and the proportion of patients lost to follow-up.

### 2.7 Statistical analysis

Categorical variables were presented as counts and percentages, and continuous variables as medians with IQRs. Group comparisons were performed using Fisher’s exact test or the chi-squared test for categorical variables and the Mann–Whitney U-test for continuous variables.

The primary analysis used a survival approach. Time-to-event was defined as the time from the day of hospital visit to the first composite event. Survival functions were estimated using the Kaplan–Meier method and compared using the log-rank test. A Cox proportional hazards model estimated the association between pathway allocation and the outcome, expressed as hazard ratios (HRs) with 95% CIs.

Although several clinically relevant covariates (ie, age, sex, active cancer, history of VTE, provoked episode, renal function, venous thromboembolic disease & bleeding score, anticoagulant type, body mass index [BMI], and thrombophilia) were initially considered, only 35 primary outcome events occurred. Therefore, to avoid model overfitting and in accordance with the rule of approximately 10 events per variable, the final multivariable Cox proportional hazards model was deliberately restricted to age and provoked VTE status. The proportional hazards assumption was verified using Schoenfeld residuals.

Given the nonrandomized design, a propensity score (PS) was estimated using logistic regression, and inverse probability of treatment weighting (IPTW) was applied.

The PS estimated each patient’s probability of enrollment in the structured pathway based on baseline covariates. Weighting was then applied to create a pseudopopulation in which measured baseline characteristics were balanced between groups.

The PS was estimated using logistic regression with age, sex, BMI, history of VTE, provoked event, estimated glomerular filtration rate, venous thromboembolic disease & bleeding score, and thrombophilia. The average treatment effect was targeted. Covariate balance before and after weighting was assessed using standardized mean differences (SMDs), with values <0.10 considered to indicate adequate balance.

Secondary analyses used logistic regression to assess loss to follow-up, reported as odds ratios (ORs) with 95% CIs. Sensitivity analyses were performed excluding patients with follow-up <3 months, analyzing each composite component separately, and using PS as a covariate. All tests were 2-sided, with *P* < .05 considered significant. Analyses were conducted in R software (version 4.3.2; R Core Team, R Foundation for Statistical Computing, Vienna, Austria).

### 2.8 Ethical considerations

This retrospective observational study was conducted in accordance with the Declaration of Helsinki and French regulations regarding retrospective research using routine care data. The study protocol was reviewed and approved by the “Comité Local d’Éthique et d’Accompagnement” of Avicenne Hospital, Assistance Publique–Hôpitaux de Paris.

All patients received written information about the study and the use of their anonymized medical data for research purposes. A nonopposition procedure was implemented, and patients were given the opportunity to refuse participation. No patient opposed participation.

Data were collected and analyzed anonymously.

Written information regarding the study and the nonopposition procedure was sent to all eligible patients using the contact information available in the hospital medical record, including patients who were subsequently lost to hospital follow-up.

## 3 Results

### 3.1 Baseline characteristics

A total of 161 patients were included: 97 (60%) in the standard pathway group and 64 (40%) in the structured pathway group. Baseline characteristics are summarized in Table 1. The 2 groups were largely comparable, though several differences were statistically significant.

**Table 1:** Baseline characteristics.

Characteristic	Overall (*N* = 161) *n* (%)^a^	Standard pathway (*n* = 97) *n* (%)^a^	Structured pathway (*n* = 64) *n* (%)^a^	*P* value
Age (y), median (IQR)	56.0 (26.0)	55 (29.0)	58.0 (20.5)	.18
Age > 85 y	7 (4.3)	4 (4.1)	3 (4.7)	>.99
Female sex	84 (52)	55 (57.0)	29 (45)	.21
BMI (kg/m^2^), median (IQR)	28.1 (9.1)	28.30 (7.6)	28.0 (8.9)	.38
BMI ≥ 30 kg/m^2^	58 (36)	36 (38)	22 (34)	.78
Creatinine (μmol/L), median (IQR)	72.5 (26.8)	71.2 (29.0)	74.5 (23.6)	.77
GFR (mL/min), median (IQR)	89.5 (26.8)	89.5 (33.5)	89.5 (27.3)	.99
CRP (mg/L), median (IQR)	3.0 (3.0)	4.0 (3.0)	3.0 (2.0)	.045
PT (s), median (IQR)	84.0 (18.0)	85.0 (17.0)	84.0 (21.5)	.89
aPTT (s), median (IQR)	0.99 (0.2)	0.97 (0.2)	1.0 (0.2)	.08
Platelets (G/L), median (IQR)	307.0 (159.0)	293.0 (152.0)	328.0 (171.8)	.34
Active cancer	8.0 (5.0)	4.0 (4.1)	4 (6.3)	.71
Thrombophilia	17.0 (11.0)	10.0 (10.0)	7 (11.0)	>.99
History of VTE	61.0 (38.0)	35.0 (36.0)	26 (41.0)	.68
Isolated DVT proximal	91 (56)	61 (67)	30 (50)	.006
Isolated DVT distal	6 (3.7)	4	2	
Isolated PE	8 (5)	1 (1.0)	7 (11)	.03
Isolated PE segmental	8 (5)	1	7	.03
Isolated PE subsegmental	0 (0)	0 (0)	0 (0)	
Combined DVT + PE	56 (34)	31 (32)	25 (39)	.4
Combined DVT + PE segmental	54 (33)	30	24	
Combined DVT + PE subsegmental	2 (1)	1	1	
Provoked thrombosis episode	32.0 (20.0)	22.0 (23)	10.0 (16)	.37
Low bleeding score (<2)	136.0 (86.0)	85.0 (89)	51.0 (82)	.35
High bleeding score (≥2)	22.0 (14.0)	11.0 (11)	11.0 (18)	.35
Aspirin treatment	4.0 (2.5)	2.0 (2.1)	2.0 (3.1)	>.99
LMWH	10.0 (6.3)	7.0 (7.3)	3.0 (4.7)	.48
DOAC	133.0 (83)	77.0 (80)	56.0 (88)	.48
VKA	17.0 (11)	12.0 (13)	5 (7.8)	.48
Patients without primary care	24 (15)	13 (13)	11 (17)	
Follow-up time (mo), median (IQR)	16.0 (13.0)	15.0 (13.0)	22.0 (9.3)	<.001

aPTT, activated partial thromboplastin time; BMI, body mass index; CRP, C-reactive protein; DOAC, direct oral anticoagulant; DVT, deep vein thrombosis; GFR, glomerular filtration rate; LMWH, low-molecular-weight heparin; PE, pulmonary embolism; PT, prothrombin time; VKA, vitamin K antagonist; VTE, venous thromboembolism.

a Unless otherwise specified.

For initial VTE presentation, patients were categorized into mutually exclusive groups: isolated DVT, isolated PE, or combined DVT and PE. Isolated DVT was more frequent in the standard care group than in the structured pathway group (65/97 [67%] vs 32/64 [50%]), whereas isolated PE was more frequent in the structured pathway group (7/64 [11%] vs 1/97 [1.0%]). Combined DVT and PE occurred in 31/97 patients (32%) in the standard care group and 25/64 patients (39%) in the structured pathway group. Overall, the distribution of initial VTE presentation differed significantly between the 2 groups (*P* = .006).

Among patients with isolated DVT, 91 had proximal lower-limb DVT, and 6 had distal DVT. Among patients with isolated PE, all events were segmental, with no isolated subsegmental PE. Among patients with combined DVT and PE, 54 had segmental PE, and 2 had subsegmental PE. The simplified PE Severity Index was not systematically available in this retrospective dataset.

The median C-reactive protein level was slightly lower in the structured pathway group (3.00 vs 4.00 mg/L, *P* = .045). Follow-up duration was longer in the structured pathway group (median, 22 vs 15 months; *P* < .001), and the proportion of patients lost to hospital follow-up was markedly lower (7.8% vs 30%; *P* = .002). Other clinical and biological variables, including age, sex, BMI, renal function, VTE history, bleeding risk scores, and anticoagulant type, did not differ significantly between the groups.

Among patients without an identified primary care physician at baseline, the care coordinator successfully identified a community general practitioner for 13 patients in the structured pathway group. In contrast, 10 patients in the standard care group remained without an identified primary care physician.

Follow-up duration differed between the 2 groups and was longer in the structured pathway group than in the standard care group (median, 22 vs 15 months, *P* < .001). Because longer follow-up may increase the probability of detecting clinical events, time-to-event analyses using Cox proportional hazards models were performed to account for differences in observation time among patients. This imbalance was also considered when interpreting the results.

### 3.2 Primary analysis

Complication-free survival differed between the structured pathway and standard care groups (log-rank *P* = .02; Figure) as in Supplementary Figure 1. In univariate Cox regression, enrollment in the structured pathway was associated with a lower risk of composite complications (HR, 0.43; 95% CI, 0.21-0.91; *P* = .03). The univariate Cox regression results are shown in Supplementary Table 1.

**Figure:**
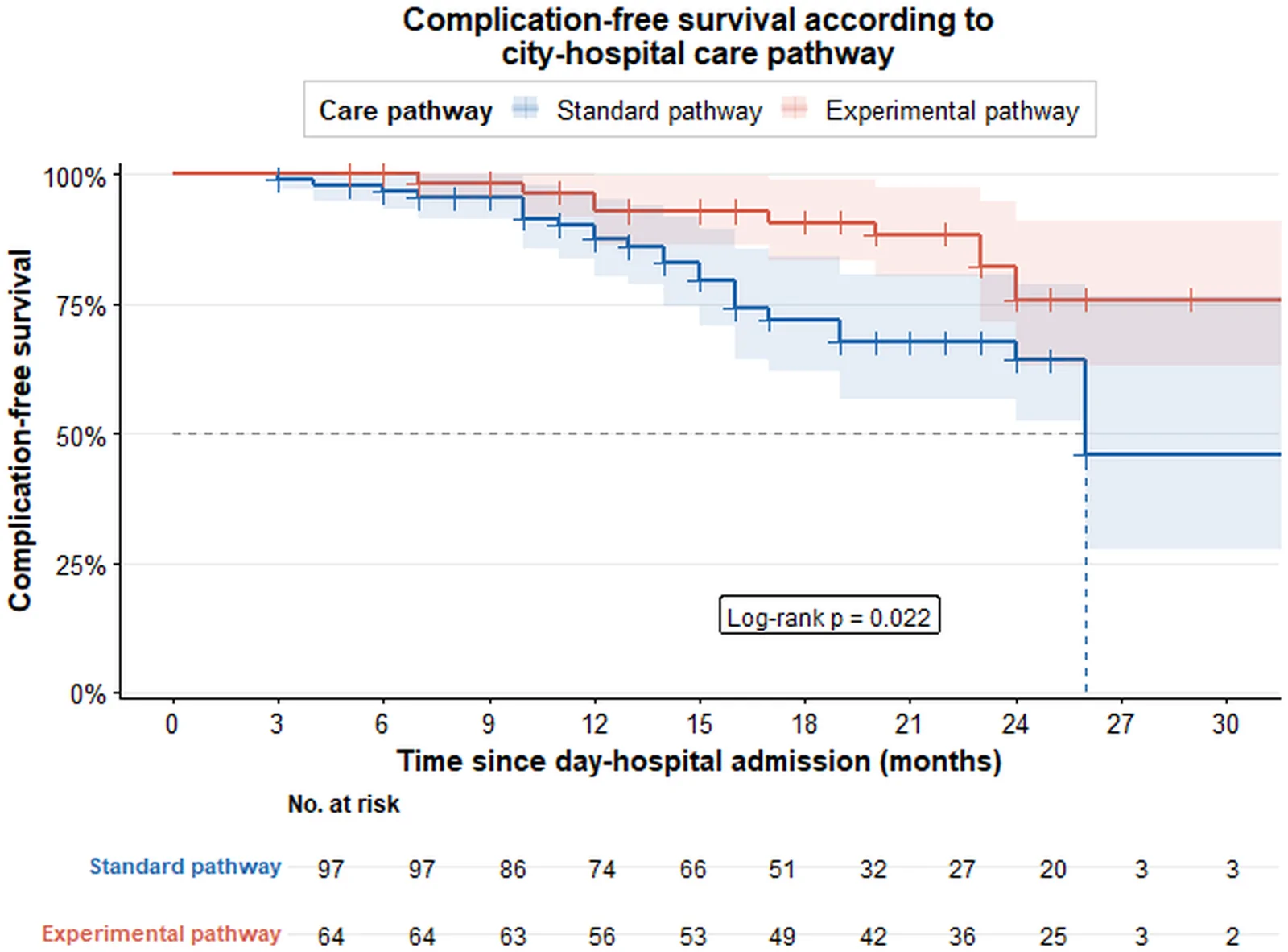
Kaplan–Meier curve comparing complication-free survival between the structured pathway and standard care groups.

After adjusting for age and provoked VTE, the structured pathway remained associated with a lower risk of composite complications (HR, 0.44; 95% CI, 0.21-0.93; *P* = .03). The proportional hazards assumption was verified (global test: chi-square = 4.18; df = 3; *P* = .24), and the corresponding Schoenfeld residual plots are shown in Supplementary Figure 3. After IPTW adjustment, all covariates achieved adequate balance (|SMD| < 0.1), and the association remained statistically significant (HR, 0.44; 95% CI, 0.21-0.92; *P* = .03). Covariate balance before and after IPTW is shown in Supplementary Figure 4. The results were consistent across univariate, multivariable, and IPTW-weighted models (Tables 2 and 3).

**Table 2:** Multivariable Cox regression analysis.

Characteristic	HR	95% CI	*P* value
Care pathway			
Standard pathway	Ref.	–	–
Experimental pathway	0.44	0.21-0.93	.03
Age (per year)	0.99	0.97-1.01	.20
Provoked VTE			
No	Ref.	–	–
Yes	1.53	0.68-3.42	.30

Active cancer was excluded due to complete separation (0 events in 8 patients).

HR, hazard ratio; Ref., reference; VTE, venous thromboembolism.

**Table 3:** Inverse probability of treatment weighting Cox regression.

Characteristic	HR	95% CI	*P* value
Care pathway			
Standard pathway	Ref.	–	–
Structured pathway	0.44	0.21-0.92	.03

Inverse probability of treatment weighting estimates used logistic regression; a robust variance estimator was applied.

HR, hazard ratio; Ref., reference.

The association between the structured pathway and the primary composite endpoint was consistent across the univariate Cox model, the parsimonious multivariable Cox model, and the IPTW-weighted Cox model, with nearly identical HRs of 0.43 to 0.44 (Table 4).

**Table 4:** Consistency of the treatment effect across models.

Model	HR	95% CI	*P* value
Univariate Cox	0.43	0.21-0.91	.03
Multivariable Cox	0.44	0.21-0.93	.03
IPTW Cox	0.44	0.21-0.92	.03

HR, hazard ratio; IPTW, inverse probability of treatment weighting.

### 3.3 Secondary analyses

Among individual outcome components, the proportion of patients with hospitalization or death was significantly lower in the structured pathway group (3.1% vs 12%; *P* = .048). Thrombotic recurrence (14% vs 20%; *P* = .40) and hemorrhagic events (1.6% vs 6.2%; *P* = .25) were numerically lower without reaching statistical significance (Table 5).

**Table 5:** Clinical outcomes by care pathway.

Outcome	Overall (*N* = 161)	Standard pathway (*n* = 97)	Structured pathway (*n* = 64)	*P* value
Thrombotic recurrence	28 (17)	19 (20)	9 (14)	.40
Hemorrhagic event	7 (4.3)	6 (6.2)	1 (1.6)	.25
Hospitalization or death	14 (8.7)	12 (12)	2 (3.1)	.048
Composite complication	35 (22)	25 (26)	10 (16)	.17

Values are *n* (%). *P* values were calculated using Fisher’s exact test.

Among the 34 patients lost to hospital follow-up, 29 (85%) were in the standard pathway group and 5 (15%) in the structured pathway group. In this subgroup, the structured pathway showed a nonsignificant trend toward fewer complications (HR, 0.38; 95% CI, 0.09-1.67; *P* = .20), though the analysis was severely underpowered. The Cox regression restricted to patients lost to hospital follow-up is presented in Supplementary Table 2.

Patients lost to hospital follow-up had a significantly higher cumulative incidence of complications than those who were followed (log-rank *P* < .001). Complication-free survival by loss-to-follow-up status is shown in Supplementary Figure 2. In multivariable analysis, after adjusting for care pathway and age, loss to follow-up was associated with a 3.5-fold higher risk of complications (HR, 3.50; 95% CI, 1.71-7.18; *P* < .001). The corresponding multivariable Cox regression is presented in Supplementary Table 3.

Enrollment in the structured pathway was associated with lower odds of loss to follow-up (OR, 0.21; 95% CI, 0.07-0.53; *P* = .002) after adjustment for age and sex. Predictors of loss to hospital follow-up are presented in Supplementary Table 4.

### 3.4 Sensitivity analyses

All prespecified sensitivity analyses supported the robustness of the primary result. Across models, the association between the structured pathway and a lower risk of complications remained consistent, with HRs ranging from 0.17 to 0.53. Detailed results of all sensitivity analyses are presented in Supplementary Table 5.

## 4 Discussion

In this single-center retrospective cohort of 161 patients with VTE managed in a day hospital setting, enrollment in a structured city hospital care pathway was associated with a lower risk of composite complications and a lower rate of loss to hospital follow-up compared with standard care.

The higher proportion of patients receiving VKAs in the standard care group is an important consideration. Because VKA therapy requires regular international normalized ratio monitoring and closer medical follow-up than DOAC therapy, this imbalance would be expected to favor the standard care group rather than the structured pathway group. Nevertheless, the structured pathway group remained associated with a lower risk of composite complications across analytical approaches.

### 4.1 Risk of VTE complications

VTE is associated with a substantial burden of complications, including thrombotic recurrence, bleeding events, hospitalization, and death. In our study, the composite complication rate was 22% overall, with 26% in the standard pathway group vs 16% in the structured pathway group. Hospitalization or death occurred in 12% of standard pathway patients compared with only 3.1% of structured pathway patients (*P* = .048).

Macchiavelli [5] highlighted that patients with VTE are at increased risk of adverse drug events and readmissions during transitions of care. The author noted that anticoagulants, particularly warfarin, account for a high proportion of emergency hospitalizations for adverse drug events in older adults, with approximately 20 hospitalizations per 10,000 outpatient visits [5]. The emergence of DOACs has improved the safety profile, but risks remain, particularly when patients are lost to follow-up or when medication reconciliation is inadequate [5].

Misky et al. [6] demonstrated that implementing a VTE clinical pathway reduced hospital length of stay from 4.4 to 3.1 days (*P* < .001) and decreased costs per VTE patient from $7610 to $5295 (*P* < .005). Notably, readmission rates declined from 9.4% to 5.6% (*P* = .25), and uninsured patients experienced dramatic cost reductions from $9953 to $4304 (*P* = .001) [6]. These findings align with our observation that structured pathways may be associated with fewer hospitalizations and complications. The consistency of results across different settings and populations supports the generalizability of structured VTE care pathways.

Kapoor et al. [7] found that among VTE patients, the combined population had 34 hospitalizations and 29 emergency department (ED) visits within 90 days, with 39.7% of encounters occurring in the first 30 days. Importantly, most hospitalizations and ED visits in the control group were unrelated to VTE, bleeding, or anticoagulation (79%), suggesting that patients with VTE have other significant comorbidities and medication issues that drive utilization [7]. Our study complements this finding by suggesting that a structured pathway addressing care coordination and follow-up may be associated with fewer overall complications, even when some complications are unrelated to VTE itself. This suggests that improving care organization may influence the clinical trajectory independently of a direct effect on biological events.

More recently, Ciemins et al. [8] conducted a type 2 effectiveness-implementation hybrid study across 6 US health systems to develop and implement targeted interventions to improve care transitions for VTE patients. They observed an increase in 7-day follow-up from 25.2% to 33.6% (*P* < .0001) and a reduction in VTE-related hospitalizations or ED visits within 45 days from 7.8% to 6.3% (*P* = .03), while maintaining low rates of anticoagulant-associated adverse drug events [8]. They found that a combination of interventions, including safer prescribing and management of anticoagulants, standardized protocols with centralized care processes, and improved care team communication, was associated with improved outcomes [8]. These findings support our approach of combining multiple interventions within a structured pathway.

### 4.2 Loss to follow-up

Our study identified loss to follow-up as a major determinant of complications. Patients lost to follow-up had a 3.5-fold higher risk of complications.

Rokosh et al. [9] reported that more than one-third (38%) of patients diagnosed with VTE were lost to VTE-specific follow-up within 3 months of initial discharge. Notably, 85% of these patients were discharged on anticoagulation, highlighting the potential risk of bleeding or recurrent thrombosis without adequate monitoring [9]. In multivariate analysis, discharge to a facility (rehabilitation, skilled nursing, or long-term care) was associated with a 95% increase in the odds of loss to follow-up (OR, 1.95; 95% CI, 1.1-3.6; *P* = .03) [9]. Our study extends this finding by showing that, in our cohort, the structured city hospital care pathway was associated with a lower rate of loss to follow-up (7.8% vs 30%). In addition, loss to follow-up was associated with a higher risk of complications.

Macchiavelli [5] emphasized that patients are often lost to follow-up or follow-up outside the time window for safe care. The author noted that when patients are discharged to another care facility (acute rehabilitation, skilled nursing facility, or long-term acute care hospital), an additional care team becomes involved, which can lead to potential information loss and reduced continuity. At every transition step, there is a risk of error, and the more steps, the greater the risk [5]. This observation aligns with our finding that structured pathways with dedicated care coordinators may help reduce these risks by ensuring continuity across transitions.

The systematic review by Kao et al. [10] found that higher COC in outpatient settings is associated with fewer hospitalizations for ambulatory care-sensitive conditions. Of the 15 studies reviewed, 93.3% showed a statistically significant association between higher COC and a lower likelihood of hospitalization. The Bice–Boxerman COC index was the most common measure used in claims data studies, and most studies measured COC before the outcome measurement period to strengthen causal inference [10]. Our study extends these findings to VTE, suggesting that a structured pathway designed to improve continuity through scheduled follow-up appointments (at 1, 3, 9, and 12 months), care coordination, and community provider engagement may be associated with lower rates of loss to follow-up and clinical complications.

### 4.3 Rationale

Several mechanisms may explain the association between structured care pathways and fewer complications among VTE patients. First, timely follow-up may enable earlier detection and management of anticoagulation-related issues, such as bleeding or inappropriate dosing. Second, care coordination between hospital and community providers may reduce medication errors and improve adherence. Third, direct communication channels may allow community providers to quickly consult hospital physicians when complications arise.

Macchiavelli [5] noted that medication reconciliation is an important process during transitions, verifying medications and documenting those started or discontinued. The complexity of medication treatments has grown, and anticoagulants can have dire consequences if taken incorrectly. Confusion about dosing schedules and self-administration can be detected by clinicians during evaluation. Without appropriate medication reconciliation, the patient is at risk of harm [5]. In our pathway, the care coordinator specifically informed community pharmacists about anticoagulant treatment and arranged home nursing visits to assess adherence and fall risk, directly addressing these vulnerabilities.

Misky et al. [6] emphasized that timely posthospital follow-up care significantly affected their pathway results. Historically, uninsured patients lacked primary care follow-up and often waited 3 months for an initial clinic visit. Through timely care coordination with local community health clinics, uninsured VTE patients were routinely scheduled for care within 72 hours. Posthospital care was further addressed through follow-up phone calls that monitored patient understanding and care and identified how and where potential medical needs were best met [6]. Our pathway incorporated similar elements, including scheduled follow-up appointments at multiple time points and direct coordination with community providers.

### 4.4 Implications for clinical practice

Our findings have several important implications for clinical practice. First, loss to follow-up should be recognized as a clinically meaningful risk factor for complications among VTE patients. Health systems should actively track follow-up rates and implement interventions to improve COC. Second, structured care pathways that include systematic follow-up scheduling, care coordination, and direct communication between hospital and community providers may help reduce loss to follow-up and complications. Third, these interventions may be particularly beneficial in medically underserved areas with poor baseline care coordination, as our pathway findings in Seine-Saint-Denis, France, suggest.

The Society of Hospital Medicine Facilitation of Anticoagulation for Safer Transitions program, described by Macchiavelli [5], provides a model for such interventions. This mentored quality improvement program includes 2-day follow-up calls, enhanced medication reconciliation, VTE order sets, oral anticoagulation readiness checklists, standardized discharge readiness checklists, and direct communication of the care plan to the next provider using a standardized script [5]. The program enrolled 1322 patients across multiple sites, with readmission rates of 3.8% and ED utilization of 4.8% [5]. These results, together with our findings, support the widespread implementation of structured VTE care pathways.

### 4.5 Strengths and limitations

This study has several strengths, including robust statistical methods (multivariable Cox regression and IPTW weighting), consistent results across multiple analytical approaches, and a focus on a medically underserved population where such interventions are most needed.

Several limitations must be acknowledged. First, because this was a retrospective, single-center, observational study, causal relationships cannot be established. Although we applied multivariable adjustment and IPTW to control for measured confounders, residual confounding by unmeasured factors (eg, health literacy, socioeconomic status, and treatment adherence) cannot be excluded. Second, the limited sample size (161 patients) reduced statistical power for secondary and subgroup analyses, particularly for individual components of the composite endpoint. Third, as noted by Rokosh et al. [9], patients were considered lost to follow-up if they did not return to our institution, although they may have received care elsewhere.

Follow-up duration was longer in the structured pathway group. Although time-to-event methods were used to account for differences in observation time, unequal follow-up duration remains a potential limitation of this retrospective study.

However, given the integrated nature of our healthcare system and the medically underserved characteristics of the population, this is unlikely to have substantially affected our findings.

## 5 Conclusion

In conclusion, this retrospective, single-center cohort study found that a structured city hospital care pathway was associated with a lower risk of composite complications and a lower rate of loss to follow-up among patients managed for VTE in a day hospital setting. These findings suggest that structured coordination between hospital and community healthcare professionals may improve COC, particularly in medically underserved areas. Further prospective studies are needed to confirm these findings and assess the generalizability of this care model.
