# Supplementary material for: Impact of an integrated hospital-community care pathway on the management of venous thromboembolism: the venous thromboembolic disease care pathway 93 study
Source: Res Pract Thromb Haemost. 2026 Sep 12;10(7):106956. doi: 10.1016/j.rpth.2026.106956 (PMC13676199; doi:10.1016/j.rpth.2026.106956)
Supplement: Supplementary file 1 [file mmc1.docx]

**Supplementary Material**

**Supplementary table**

**Supplementary table S1 : Full univariate Cox régression analysis**

**Supplementary table S2 : Cox regression among patients lost to hospital follow-up**

**Supplementary table S3: Association between loss-to-follow-up status and composite complications**

**Supplementary table S4 : Logistic regression analysis of predictors of loss to hospital follow-up**

**Supplementary table S5 : Sensitivity analyses for the association between the structured pathway and composite complications**

**Supplementary figure**

**Supplementary figure S2 : Complication-free survival according to loss-to-follow-up status**

**Supplementary figure S3 : Schoenfeld residuals assessing the proportional hazards assumption**

**Supplementary figure S4 : Covariate balance before and after IPTW weighting**
